# Insomnia: Especially in Its Relationship to Dyspepsia
*A lecture delivered at the London School of Clinical Medicine.


**Published:** 1913-08-30

**Authors:** Guthrie Rankin

**Affiliations:** Physician to the *Dreadnought* and Royal Waterloo Hospitals.


### INSOMNIA: ESPECIALLY IN ITS RELATIONSHIP TO DYSPEPSIA.*

By GUTHRIE RANKIN, M.D., F.R.C.P., Physician to the *Dreadnought* and Royal Waterloo Hospitals.

Insomnia is a word which means literally want of sleep, but it has come to be applied popularly to all degrees of disturbance of the night's rest.

That mysterious condition of repose in which our consciousness is in abeyance and which we call sleep has ever been a subject of interest and investigation, but we are still only in the twilight of knowledge in regard to its true nature or the ultimate physical conditions upon which it depends. Cerebral anæmia seems to be an important factor, and this is probably associated with exhaustion of the brain and chemical changes due to an accumulation of fatigue products, with consequent diminished receptivity for afferent stimuli. We are aware that sleep may be disturbed by a multitude of causes, but in its perfect expression we only know it as "Nature's sweet restorer," upon which we all depend absolutely for health and happiness. A hard-and-fast rule cannot be applied to indicate the amount of sleep required by every individual in order to secure complete restoration of nervous energy. No two persons are similarly constituted, for whereas one is refreshed and reinvigorated by four or five hours' repose, another requires seven or eight hours to achieve a like result. Sex, age, temperament, occupation, habits, climate, and environment are all factors of importance which bear upon the faculty of sound sleep, and as each of these conditions is of individual application, it follows that the personal equation makes any general rule impossible of universal application.

In all cases of insomnia there are two points to be carefully inquired into—namely, the family record as regards stability of nervous system and the personal record as regards early training. Certain individuals come of a stock none of whom has ever known what it was to sleep soundly; others belong to a family in which, throughout some generations, various forms of neuroses or eccentricities can be traced; while others again own an ancestry in which gout or some other metabolic infirmity has been such a predominant event that tendencies to waste-retention may be fairly assumed as a general inheritance.

No less important is it to know something of the person's upbringing, whether he was nursed by his mother or artificially reared; whether at a later period questions of exercise, rest, clothing and food were wisely regulated; whether later still his education was prudently adapted to the demands upon his strength of growth and development; and whether, when puberty approached, he was taught to know something of the sexual side of his nature and judiciously warned against the evils of a surrender, in any form, to their insistent demands.

Sleep disturbance depends upon many circumstances in ordinary life when the health is apparently robust, but in its more serious manifestations sleeplessness is mostly related to conditions of disease in which the normal balance of organic or functional activity is interfered with.

Apart from pain and organic disease, perhaps the most common causes of insomnia are overwork and worry. The one condition like the other leads to the accumulation of an excess of waste in the body, and upon this there ensues, in time, disturbed digestion, nervous irritability, mental depression, and sleeplessness. In those who are so predisposed one of the earlier consequences of overwork and worry is neurasthenia, and in this unstable state of the nervous system, as we all know, sleep-disturbance is one of the most depressing associations. In the milder varieties of neurasthenia it takes the form of light and easily disturbed slumber, which is often interrupted by dreams mostly bearing upon the events of the preceding day. In more advanced stages of the disease insomnia may become the most clamant symptom, all attempts at sleep being futile without the aid of some form of sedative. Next to work and worry, and often associated with either or both, some form of disturbed digestion is responsible for many restless and sleepless nights.

Insomnia, which accompanies all painful and pyrexial illness, becomes part of the general disturbance of health, and its treatment is merged in the management of the foundational conditions.

What I desire specially to direct attention to is that variety of insomnia which is not caused by nor dependent on a passing storm of acute disease, but which is a chronic condition induced by the wear and tear of everyday life. I need hardly remind you that there are many avenues through which strain may invade the citadel of health. Poverty, grief, disappointed ambitions, unfulfilled hopes are etiological factors no less potent than physical overwork, mental stress, or functional excess. It is obvious that the first step towards the restoration of normal sleep is to ascertain the underlying cause upon which its aberration depends and by correcting that fault, whatever it be, to restore Nature to her proper rhythmic balance. But though theoretically this is the sound scientific principle by which we should be guided, we all know as practical men how difficult, and in many instances how impossible, it is to carry out this primary indication. We can at best hope by tactful management and patient endeavour to lead the sleepless errant gradually back to the comfort of physiological integrity by re-educating him to a better sense of proportion between income and outgo whether mental or physical; by strengthening him where he is weak in this, that, or the other function of his organic health; and by overcoming the depression from which he always suffers by a wholesome preaching of the gospel of hopeful resignation. It

* A lecture delivered at the London School of Clinical Medicine.

is perfectly true that no counsels of perfection will at first prevail in subduing a proud spirit accustomed to affluence and luxury, to the hardships and trials of an unexpected and too often undeserved advent of poverty; that no doctrine of the inestimable value of simplicity in habits of life will at first sight persuade the *bon-viveur* that his salvation depends upon abstinence; that no eugenic theory will exorcise like a gifted talisman the chagrin and bitter disappointment of a straying son or daughter who has disappointed sadly, and perhaps irrevocably, parental hopes and ambitions; that no system of philosophy in the world will immediately enable the victim of a deep-seated mental depression engendered by the crushing circumstances of ill-health or worldly misfortune to see the proverbial silver lining behind the lowering shadow of his despair. But though, under those and other similar circumstances, miraculous cures are unlikely, the patient insistence upon the principles of submission and compensation seldom fail to pave the way for an ultimate reconciliation of personal desire to the necessities, however hard they may be, of a stern and relentless fate. All this is true so far as the mental aspect of these cases of sleeplessness is concerned, but the mental state is one side of the picture only; there is almost invariably a physical side as well, which manifests itself in distinctive disturbance of the digestive organs. Whether this digestive disorder is to be looked upon as cause or effect it is difficult to say, but it seems almost inevitable that metabolism must be influenced by any condition which so upsets the general economy as to encroach upon the resting-time requisite for the recuperation of spent energies, and it is not unlikely that, in most cases, a vicious circle becomes established whereby digestive faults are accentuated by insomnia to which in their turn they contribute materially.

### Two Varieties of Dyspepsia.

From all this it follows that in most of the cases of chronic insomnia with which we meet, the first direction our inquiries should take is towards the condition of the digestive system, and our earliest efforts at treatment must be towards establishing it upon a basis of stability and normal functioning.

Apart from the indigestion which is symptomatic of organic disease in the stomach or elsewhere, there are two varieties of the functional type of dyspepsia which are closely associated with neurasthenia on the one hand and with insomnia on the other, and which, when present, require to be dealt with before any hope can be entertained that either the neurasthenia or the insomnia by which they are accompanied can be cured.

The one type, which may be conveniently designated the asthenic type of indigestion, is dependent upon a want of power in the stomach as regards both secretion and contractility, and is met with in people, mostly women, who are badly nourished, pale, thin, and run down from improper or insufficient food, irregularity of habits of everyday life, often accompanied by excess of mental or physical work, or by the drain upon the strength of some exhausting discharge. They complain of loss of appetite, flatulent distension and stomach discomfort immediately after taking food; eructations that are sometimes sour and often offensive; a fear of food because of its effect in increasing discomfort, and a desire to relieve the situation by emesis; a constant sense of weariedness and mental depression; irregularity of bowels, diarrhœa alternating with constipation; and persistent insomnia or at best short unrefreshing and dream-troubled sleep. On physical examination such a patient presents a flabby teeth-indented and coated tongue; the teeth are often carious and not sufficiently well looked after; the complexion is pale and bears that muddy look so suggestive of toxic absorption; the heart is feeble and hæmic murmurs are common incidents; the stomach is tender to touch all over and is of normal size, or in advanced cases in a condition of atonic dilatation; its contents contain less hydrochloric acid than normal. The bowels are more or less distended and present active peristaltic movements accompanied by distressing borborygmal sounds. The colon is frequently tender to touch over the cæcum and at the flexures, and is often found to be loaded with fæces.

It is not uncommon to discover a displacement of the right kidney accompanied, in some instances, by a sagging of all the abdominal contents, which causes the patient to experience a wearying sense of bearing-down and a desire to obtain relief by assuming the recumbent position on every convenient opportunity.

The other, or sthenic type, more common in men than in women, presents a striking contrast to that just described. The patient is often well nourished, and may be even plethoric; he complains of pain in his stomach, which comes on three or four hours after a meal, is usually preceded by heartburn and pyrosis, and is always relieved by food or an alkali, such as bicarbonate of soda. He retains a good appetite, and has but little inclination to sickness. His sleep at night is uncertain, and is often disturbed at about three in the morning by an attack of hunger-pain, against which he learns to provide by having food of some kind always at his bedside. His bowels are, as a rule, confined, and he is depressed about himself and curiously introspective. On physical examination his tongue may be coated, but is more frequently clean and almost irritable-looking; his teeth may require attention, but they are not so constantly in a state of neglect as in patients suffering from the asthenic type of the disorder. His heart is usually vigorous in action and without suspicion of murmur, though frequently there is irregularity of rhythm and some accentuation of the second aortic sound. The stomach may be dilated, often down to the level of the umbilicus, due partly to enfeeblement of the muscular wall and partly to spasm of the sphincters at the cardiac and pyloric orifices; to the right of the epigastrium and just over the situation of the pylorus and duodenum there is localised tenderness, which is intensified by deep pressure and associated with a marked protective contraction of the right rectus muscle. The stomach contents are abnormally acid from an excess of hydrochloric acid, and also of organic acids produced by fermentative processes. The lower abdomen is normal, but fæcal masses can be felt sometimes in the colon, and there may be tenderness over the cæcum.

Both these varieties of dyspepsia are liable to become aggravated by fermentative processes, which seriously add to the discomfort of the sufferer, because they rapidly produce vertigo, vomiting, offensive eructations, increased dilatation of the stomach, tympanitis, pains in the limbs, headaches, and increased evidences of toxic absorption.

You will observe that the clinical picture in cases of the sthenic type closely resembles that met with in patients who are suffering from duodenal ulcer, but in the purely functional cases there is no history of either hæmatemesis or melæna. Much confusion has arisen in the differentiation between sthenic dyspepsia and duodenal ulcer, and attention has been drawn frequently to the need for care in arriving at a firm diagnosis until the effect on the symptoms of suitable medicinal and dietetic treatment has been carefully watched and recorded. It is quite certain that what has been called "hunger-pain" is in no sense pathognomonic of duodenal ulceration. Though it undoubtedly occurs as one of the evidences of such a lesion, it is an equally marked feature of the type of indigestion we are now considering.

### Dietetic Treatment.

The insomnia which accompanies these two varieties of disturbed digestion is often the symptom which more than any other drives the patient to the doctor. It is entirely a secondary development, and its relief can only be achieved by correcting the functional faults in the digestive tract. This can often be successfully accomplished by the following plan of management.

In asthenic cases the object to be achieved is stimulation of the feeble gastric functions. Milk should be given sparingly, because it delays hepatic activity and diminishes gastric secretion. Bread should be taken in limited amount and must always be stale or toasted. Vegetables are best supplied in the form of purée. Sugar is to be avoided, and may be replaced by saccharine or saxin. Condiments, such as pepper, mustard, chutney, etc., are relished, and serve to stimulate the appetite. Red meat, in its more easily digested forms and no more than cooked, should be taken at the middle-day meal, and repeated at night when circumstances do not permit of its replacement by fish or chicken. An infusion of malt with each meal is useful, and a limited quantity of stimulant twice a day may be allowed.

As regards medicine, each meal should be preceded by a simple bitter alkaline mixture such as the following: Bicarbonate of potash, twenty grains; tincture of nux vomica, ten minims; tincture of ginger, twenty minims; compound infusion of gentian to one ounce. If flatulence and acid eructations occur they will be rapidly relieved by a powder consisting of twenty grains each of carbonate of bismuth, carbonate of magnesia, and bicarbonate of soda. The bowels must be regulated by an evening pill, arranged somewhat as follows: Leptandrin and grey powder, of each one grain; aloin, one quarter of a grain; extract of belladonna, one sixth of a grain; extract of rhubarb, two grains. When the subjective symptoms have become controlled, the alkaline mixture before food may be advantageously replaced by a more tonic prescription taken after meals. One or other of the following formulæ will be found useful:—(*a*) Dilute hydrochloric acid, fifteen minims; pure carbolic acid, two grains; solution of strychnine, five minims; chloroform water to one ounce; (*b*) Finkler's papain, two grains; salol, five grains; strychnine, one thirtieth of a grain; and extract of cannabis indica, one quarter of a grain: to be dispensed in a capsule.

In sthenic cases, the treatment must be essentially sedative. It should aim primarily at counteracting the hyperacidity which is the chief disturbing factor. It is a good plan, where possible, to confine the diet entirely for three days at the commencement of treatment to milk. Following on this preliminary restriction the regimen may be made more varied, but arranged upon such a plan that sugar and starches are left out. Tea should be avoided, and red meat taken very sparingly. Condiments and alcohol are undesirable. The main difficulty in regard to the withdrawal of starch is to find a substitute for bread; but, for those who can afford it, there are now on the market many varieties of starchless, or nearly starchless, biscuits and rolls from which a choice may be made. Underground roots and farinaceous substances of all kinds should be left alone, and if bread must be taken it should be torrefied and in very small amount.

Medicinally each meal should be preceded by a sedative mixture of the following type: Tincture of belladonna and tincture of opium, of each ten minims; solution of chloride of adrenalin, twenty minims; spirits of peppermint to one drachm: to be taken in one tablespoonful of water quarter of an hour before the meal. This combination of drugs serves to control the glandular secretion of the stomach and diminishes the excitability of the mucous membrane. It does not, however, ensure an absence of hyperacidity later on; when this occurs it should be counteracted by one of the bismuth, magnesia, and soda powders referred to previously.

The bowels should be regulated by one ounce of liquid paraffin at bedtime, assisted, if need be, by a sufficient dose of Carlsbad salts before breakfast the following morning. In sthenic cases, an occasional dose of calomel is desirable. In all patients suffering from dyspepsia—no matter what its type—special care must be taken to make and keep the buccal cavity as clean and wholesome as possible.

I have dealt at some length upon this question of the treatment of dyspepsia, because a disturbed state of the digestive processes either in the sthenic or asthenic direction is such a common cause of insomnia. Under these circumstances, no permanent relief can come to the sleepless condition until the blood has been cleared of the toxic refuse of a disturbed metabolism. Many people who become the lifelong slaves of hypnotics and sedatives would be spared the acquisition of an evil and harmful habit if their earlier sleepless troubles were dealt with by correcting the digestive faults upon which their disturbed nights so often depend.

Pending the restoration of the digestive functions to a more normal condition, and in other cases where the insomnia is apparently not directly attributable to toxæmic influences emanating from the alimentary canal, no end of expedients have been suggested to induce that placidity of circulation and quietude of cerebral centres upon which sound slumber depends. It is, however, pretty certain that, apart from the pain of organic disease, interference with the normal rhythm of sleep is, in the large majority of instances, the direct result of a toxæmic process, which, though most frequently originating in the intestinal tract, may also arise from other and more obscure disturbances of the internal secretions of the body.

*(To be continued.)*

